# Use of the HRM Method in Quick Identification of FecX^O^ Mutation in Highly Prolific Olkuska Sheep

**DOI:** 10.3390/ani10050844

**Published:** 2020-05-14

**Authors:** Grzegorz Smołucha, Katarzyna Piórkowska, Katarzyna Ropka-Molik, Jacek Sikora

**Affiliations:** 1 Department of Animal Molecular Biology, National Research Institute of Animal Production, Krakowska 1, 32-083 Balice, Poland; katarzyna.piorkowska@izoo.krakow.pl (K.P.); katarzyna.ropka@izoo.krakow.pl (K.R.-M.); 2 Department of Sheep and Goat Breeding, National Research Institute of Animal Production, Krakowska 1, 32-083 Balice, Poland; jacek.sikora@izoo.krakow.pl

**Keywords:** HRM, sheep, Olkuska, *BMP-15*, prolificacy

## Abstract

**Simple Summary:**

According to the available literature, high prolificacy in sheep breeds could be caused by differences in many genes (polygenic trait) or as in some sheep breeds to a difference in one gene such as *BMP-15*. This paper describes the use of the High-Resolution Melting method in quick identification of the known mutation in the *BMP-15* gene, which affects high prolificacy in the Olkuska breed of sheep.

**Abstract:**

Olkuska is a highly prolific sheep breed in Poland. Thanks to earlier identification of the genetic basis of its prolificacy, a mutation in the *BMP-15* gene, we can use molecular biology tools to identify this causative mutation affecting prolificacy. In our research, we used the High-Resolution Melting (HRM) and Sanger sequencing methods to identify the genotypes of the studied animals. The result obtained by the HRM method is identical to those obtained by the sequencing method, which confirms the effectiveness of the HRM method and the possibility of quick and cheap identification of individuals with a FecX^O^ mutation.

## 1. Introduction

Sheep play an important role in modern agriculture. Moreover, differences in reproduction trait between breeds of *Ovis aries* allows the use of this specie as a model for research into biological process in mammals which is reproduction. Reproductive traits are especially crucial for sheep production because they determine the profitability of breeding [1,2,3]. They are characterized by low heritability, and selection based on phenotypic values is lengthy and inefficient. Genotype-based animal selection appears to be much more effective to improve prolificacy in sheep [1,4,5]. A number of prolificacy genes such as *BMPR-1B* (bone morphogenetic protein receptor 1B), *BMP-15* (bone morphogenetic protein 15), *GDF-9* (growth differentiation factor 9) and *B4GALNT2* (beta-1,4-N-acetyl-galactosaminyl transferase 2) have been identified in sheep. Although to date, many mutations increasing sheep prolificacy have been identified in various breeds and various genes [6] around the world, new genetic variants need to be recognized for some highly prolific breeds of sheep [7]. Oocytes play the primary role in controlling ovarian folliculogenesis, from the early stages up to ovulation via secretion of multiple factors critical for reproduction. The most important proteins produced and released by oocytes are Bone Morphogenetic Protein 15 (BMP15) and Growth and Differentiation Factor 9 (GDF9) belonging to transforming growth factor-beta (TGF-β), superfamily. They play an integral role in the ovulation process by affecting granulosa cell proliferation, regulating cumulus cell function in the periovulatory period and inhibiting FSH-induced granulosa cell differentiation [8]. It has been shown that different mutations in *BMP-15* and *GDF-9* genes influence ovulation rate (OR) and follicular growth [9,10]. To date, nine mutations in the *BMP-15* gene and four in the *GDF-9* gene affecting ovulation rate have been found in different sheep breeds [1,6,11]. They have different effects on reproduction performance and are inherited according to different models. Some of them increase the number of ovulating follicles in heterozygous individuals, and some cause infertility in homozygous animals in which the ovaries do not develop properly [12].

**Figure 1:**
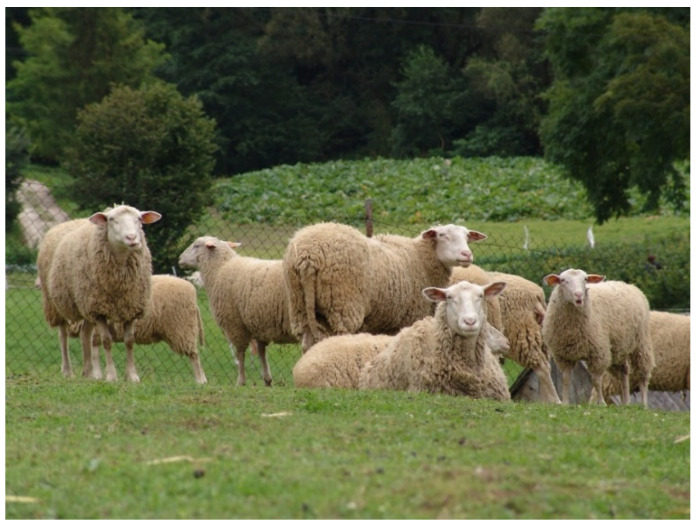
Olkuska sheep breed (photo by Jacek Sikora).

The Olkuska breed (Figure 1) is a high prolificacy (averages 200%; prolificacy is defined as the number of live offspring per parturition) sheep with excellent maternal abilities. A genomic study carried out by Demars et al. [7] and Kaczor [13] showed that high reproductive performance in Olkuska sheep is caused by a single mutation c.A1009C (N69H) in the *BMP-15* gene, named FecX^O^, which increases the ovulation rate and litter size in ewes with FecX^O^ mutation on both alleles. The mutation results in an asparagine to histidine change in the amino acid sequence of the mature BMP15 protein. As a result, the polarity and molecular weight of BMP15 have been disturbed, which influences the three-dimensional structure and alters BMP15 signaling reducing its activity by half [7]. The FecX^O^ mutation increases ewe prolificacy by 0.7 lambs in heterozygous ewes and 1.07 lambs in homozygous recessive ewes [7,13,14]. The occurrence of two mutated alleles of FecX^O^ does not cause sterility like in other mutations identified in the *BMP-15* gene, which makes it so valuable in sheep breeding [7,14]. Nowadays, due to fast-developing molecular biology techniques, the identification of gene mutations is not laborious. The FecX^O^ in Olkuska sheep was detected using Sanger Sequencing and TaqMan probe methods, which still are expensive. Therefore, in the present study, we propose a low cost, quick and sensitive High-Resolution Melting (HRM) method for identification of the FecX^O^ mutation in Olkuska sheep, which can increase the attractiveness of molecular tools in sheep breeding program and allow for more accurate selection of animals for breeding. HRM analysis is a method used for identification of genetic mutations in nucleic acid sequences. This technique is based on PCR reaction with presence of fluorescence dye that binds to double-stranded DNA (dsDNA). When dye is unbound it is shows a low level of fluorescence compared to dye bound to dsDNA when the fluorescence level increases greatly. After PCR reaction, the amplicon (usually 50 to 500 bp in length) is denatured gradually (this step is called melting analysis) from 65 °C to 95 °C with ramping by 0.2 °C. During this stage, the amplicon releases the fluorescent dye, allowing its detection. Amplicons from different samples with sequence changes (even differing by one base pair) will release fluorescent dye at different temperatures. Comparison with a reference control sample allows the sample to be assigned to the correct genotype group. The HRM method can discriminate DNA sequences based on their nucleotide composition, length, GC content, or strand complementarity and allows identification of deletions, insertions, and substitutions, as well as the detection of SNP (single nucleotide polymorphism) [15].

## 2. Materials and Methods

### 2.1. Material

Blood samples for the analysis were obtained from 100 randomly selected female sheep of the Olkuska breed from five different flocks (20 animals from each flock). 

Blood was collected from the jugular vein into 10 mL Vacutainer tubes with EDTA and stored at −20 °C prior to DNA isolation. Animal procedures were approved by the Local Animal Care Ethics Committee No. II in Kraków—permission number 1293/2016 following EU regulations.

### 2.2. Methods

#### 2.2.1. DNA Extraction

DNA isolation was performed using the A&A Biotechnology Sherlock AX reagent kit (Gdynia, Poland), according to the protocols provided by the manufacturer. Sample DNA concentration and purity were quantified using a Nanodrop 2000 spectrophotometer (Thermo Fisher Scientific, Waltham, MA, USA). All DNA samples were normalized to 100 ng/µL. 

#### 2.2.2. HRM Analysis

A Quant Studio 7k Flex Real Time PCR System (Thermo Fisher Scientific, Waltham, MA, USA) was used in HRM analysis. Reaction was carried out using MeltDoctor™ HRM Master Mix (Thermo Fisher Scientific, Waltham, MA, USA); in detail 5 µL MeltDoctor™ HRM Master Mix, 0.5 µL Primer mix (10 pM each) (Table 1), 3.5 µL PCR grade water, 1 µL DNA (100ng/µL). Primers for HRM and Sanger sequencing analysis were designed in Primer 3 software (http://www.bioinformatics.nl/cgi-bin/primer3plus/primer3plus.cgi) based on the *BMP-15* ovine reference sequence (NCBI accession number: NC_040278). HRM curves were normalized, and the genotype was assigned based on the shape of the HRM curve with the use of the High Resolution Melting software (Thermo Fisher Scientific, Waltham, MA, USA) and by visual examination. Three samples of known genotype are required for automatic genotyping so for this purpose, we selected three samples with AA, AC, CC genotypes that were previously sequenced and had a known genotype and those samples were used as references samples.

#### 2.2.3. BMP-15 Fragment Gene Sequencing

The Sanger sequencing of the *BMP-15* gene fragment was used for validation of the HRM method. Before Sanger sequencing, the PCR reaction was performed in 25 μL volume containing: 12 µL of PCR-grade water, 2.5 µL of PCR buffer with 15 mM MgCl_2_ (QIAGEN, Hilden, Germany), 5 µL of Q-Solution (5×; QIAGEN, Hilden, Germany), 3 µL of 10 mM dNTPs (APPLIED BIOSYSTEMS, Foster City, CA, USA), 0.25 µL of primer mix (each 100 pmol/µL) (Table 1), 0.25 µL of HotStartTaq DNA polymerase (5 U/µL, QIAGEN, Hilden, Germany) and 1 µL of DNA (100ng/µL) isolate. PCR thermal program: 15 min of initial denaturation at 95 °C, 35 cycles of denaturation at 95 °C for 30 s, annealing at 61 °C for 59 s and primer extension at 72 °C for 120 s., the final extension at 72 °C for 10 min. The PCR product was purified using EXOSAP-it enzyme (Affymetrix, Santa Clara, CA, USA) and sequenced from both complementary strands using the Big Dye Terminator v3.1 sequencing kit (Thermo Fisher Scientific, Waltham, MA, USA). The *BMP-15* sequence detection was performed on 3500 × l Genetic Analyzer (APPLIED BIOSYSTEMS, Foster City, CA, USA). 

#### 2.2.4. Statistical Analysis

The Hardy–Weinberg equilibrium was tested using the Court Lab-HW calculator (Michael H. Court (2005–2008) and Chi-square test using http://quantpsy.org/chisq/chisq.htm. 

## 3. Results

**Figure 2:**
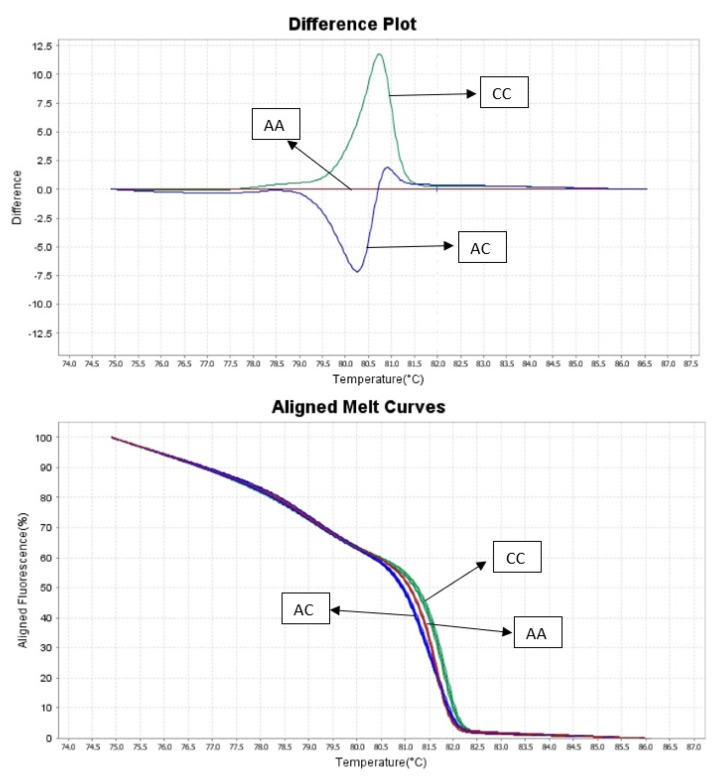
HRM genotyping results.

**Figure 3:**
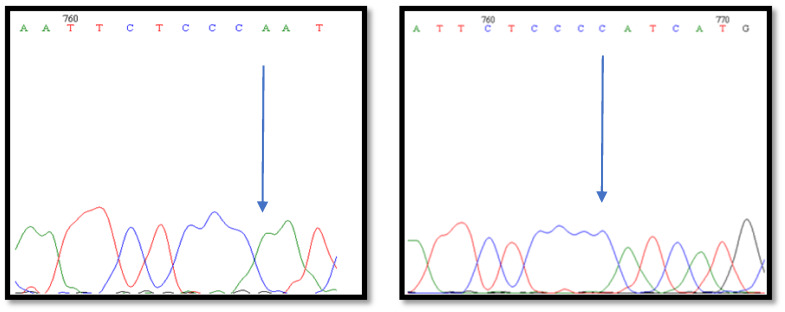
Fragments of chromatograms with the SNP mutation c.1009A > C. The polymorphic sites are marked with an arrow.

**Figure 4:**
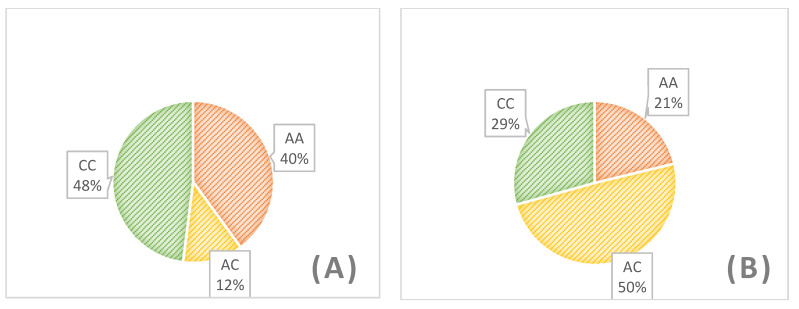
The number of genotypes observed (**A**) and expected (**B**) in the analysed population of Olkuska sheep.

For all 100 samples we obtained results from HRM analysis (Figure 2). Using control samples with known genotypes we were able to use the HRM method to automatically genotype samples. To validate the results obtained from HRM analysis all 100 samples were sequenced (Figure 3). All results obtained from two methods had the same genotype. Table 2 and Figure 4 revealed that only one flock is in Hardy-Weinberg equilibrium in the population for this locus. The allele C was more frequent than allele A in three of the analyzed flocks and its frequency varied between 0.47 to 0.67. These findings suggest that the FecX^O^ mutations is in linkage with other reproductive traits and under selective pressure for improvement of prolificacy. The selection was probably previously conducted based only on phenotypic observations however, A high frequency of the A allele indicates there is still selective potential. Differences in genotype distribution between analyzed Olkuska flocks based on the Chi-square test are presented in Table 3. One analyzed flock showed the opposite frequency of both alleles, which could suggest that in this flock the other features were prioritized for selection. 

## 4. Discussion

The identification of prolificacy genes and their effects on reproduction traits in farm animals, including small ruminants, is considered a first step towards the improvement of reproductive traits [16]. In the present study, we focus on a rapid, low-cost HRM method which allows identification of a known FecX^O^ (allele C) mutation increasing prolificacy in Olkuska sheep. Compared with the other methods (Sanger sequencing, TaqMan probes), HRM is characterized by high technical accuracy, fast detection, low cost and easier identification of results making it highly useful to automatically detect SNP c.A1009C in the *BMP-15* gene. 

HRM is a method that has many advantages but also some disadvantages and limitations. 

Thanks to the various limitations of this method described in the available literature [17,18,19], we could avoid them and carry out the experiment correctly at the design stage. DNA quality is one of the first and fundamental limitations affecting the sensitivity and reliability of the HRM method. In our experiment, we used DNA isolated from blood, which gives a good starting base for the quality and quantity of DNA obtained. Each sample was isolated using one method, normalized to the same concentration and diluted with the same buffer. This approach is consistent with that described by Słomka et al. [17] in which the author analyzed the impact of DNA quality on genotyping efficiency by the HRM method. When using other methods such as TaqMan probes and Sanger sequencing an efficient DNA isolation method and standardization of its concentration is also recommended and can have an impact on the results. 

Another important factor affecting HRM analysis is the correct design of primers. Primers should be prepared in accordance with accepted principles of primer design (a similar melting temperature for all primers, optimally 55–65 °C; avoiding secondary structures like hairpins, homodimers or heterodimers) [17]. It should also be remembered to normalize the concentration of primers before starting HRM analysis. Moreover, normalization procedure is also required using TaqMan probes as well as Sanger sequencing and influences the results. The cost of producing probes in the TaqMan method is much more expensive than the usual primers used in the HRM method, which ultimately impacts the total cost of conducting analyses. Features such as cost, speed, and quality of results are the basic criteria taken into account by breeders when choosing among available analyses. 

Another important factor often discussed in the literature is the length of the amplicon. Products larger than 400 bp may have lower sensitivity than smaller amplicons. A sufficient amplicon length may be between 80 and 250 bp for routine analysis [15]. Technical problems and reproducibility of results are extremely important factors in any method [17]. In the HRM method, the reaction is carried out in one tube, which significantly reduces the possibility of contamination, as well as reduces the risk of errors by the operator. In the case of the Sanger sequencing reaction, the results obtained are a component of many stages (PCR reaction, enzymatic cleaning up of PCR product, sequencing reaction, capillary electrophoresis), which affects the final results in the form of a chromatogram. Although in our analysis we did not experience many of the problems described in the literature, we should consider the multitude of potential problems that may occur during the analysis. However, they do not affect the attractiveness of using the HRM method. 

Comparing the HRM and Sanger sequencing detection methods for SNP analysis, the HRM method is characterized by about 60% lower cost and 80% shorter analysis time, which gives significant advantages for practical use.

The FecX^O^ mutation can be used to improve the reproductive capacity of Olkuska sheep, and the proposed detection method has potential utility in large-scale sheep breeding because it is cheap and not labor intensive. Genotyping of sheep carrying a FecX^O^ mutation is useful not only in ewes but also in rams. Due to the fact that the FecX^O^ mutation is located on the X chromosome [20], each daughter will inherit the high prolificacy allele from her father. The study of genes associated with reproductive performance in sheep is crucial and can support breeding programs as selection markers for increasing production efficiency, reducing time and cost of procedures and, thus, accelerating genetic progress due to selection.

## 5. Conclusions

In conclusion, we recommend the HRM method, using the protocol developed in the present study, in Okulska sheep breeding programs. HRM is a rapid and cheap method for identification of the FecX^O^ mutation in commercial flocks.

## Figures and Tables

**Table 1:** Primers used in Sanger sequencing and HRM analysis.

Name	Sequence [5′–3′]	Analysis Type	PCR Product Length (bp)
BMP-15F	CAGAAGACCAAACCTCTCCCTA	Sanger sequencing	498
BMP-15R	CTGATTACGCCAGTTTGCAC		
FecXO-F	TCCACCCTTTTCAAGTCAGC	HRM-PCR	248
FecXO-R	ACTCCCATTTGCCTCAATCA		

**Table 2:** The frequency of genotypes and alleles at the locus of the *BMP-15* gene and *p*-values for deviation from the Hardy–Weinberg equilibrium (HWE).

Olkuska Breed	Genotype			Allele		HWE *p*-Value
	AA	AC	CC	A	C	
Flock 1 (*n* = 20)	0.2	0.3	0.5	0.35	0.65	0.12764
Flock 2 (*n* = 20)	0.5	0.05	0.45	0.525	0.475	0.000057
Flock 3 (*n* = 20)	0.3	0.1	0.6	0.35	0.65	0.000484
Flock 4(*n* = 20)	0.3	0.05	0.65	0.325	0.675	0.000074
Flock 5 (*n* = 20)	0.7	0.1	0.2	0.75	0.25	0.001040
Total (*n* = 100)	0.4	0.12	0.48	0.46	0.54	0.0000001

**Table 3:** Differences in genotype distribution (*p*-values) between analyzed Olkuska flocks (ns- non significant).

Flocks	1	2	3	4	5
1	*	0.04	ns	ns	0.006
2		*	ns	ns	ns
3			*	ns	0.01
4				*	0.01
5					*

* means that I can not compare the same flock. 1 to 1 is marker *, 2 to 2 the same situtation etc.

## Data Availability

Source data regarding prolificacy will be made available upon request of the corresponding author.
